# Display of porcine epidemic diarrhea virus spike protein B-cell linear epitope on *Lactobacillus mucosae* G01 S-layer surface induce a robust immunogenicity in mice

**DOI:** 10.1186/s12934-024-02409-x

**Published:** 2024-05-21

**Authors:** Bin Zhang, Hongchao Gou, Haiyan Shen, Chunhong Zhang, Zhicheng Liu, Nile Wuri, Jingjing Nie, Yunzhi Qu, Jianfeng Zhang, Letu Geri

**Affiliations:** 1 College of Veterinary Medicine, Inner Mongolia Agricultural University, Hohhot, 010010 China; 2 Key Laboratory of Livestock Disease Prevention and Treatment of Guangdong Province, Institute of Animal Health, Guangdong Academy of Agricultural Sciences, Guangzhou, 510640 China

**Keywords:** PEDV, Epitope, SLP, *L. mucosae* G01, Probiotics surface

## Abstract

The Porcine epidemic diarrhea virus (PEDV) presents a substantial risk to the domestic pig industry, resulting in extensive and fatal viral diarrhea among piglets. Recognizing the mucosal stimulation triggered by PEDV and harnessing the regulatory impact of lactobacilli on intestinal function, we have developed a *lactobacillus*-based vaccine that is carefully designed to elicit a strong mucosal immune response. Through bioinformatics analysis, we examined PEDV S proteins to identify B-cell linear epitopes that meet the criteria of being non-toxic, soluble, antigenic, and capable of neutralizing the virus. In this study, a genetically modified strain of *Lactobacillus mucosae* G01 (*L.mucosae* G01) was created by utilizing the S layer protein (SLP) as a scaffold for surface presentation. Chimeric immunodominant epitopes with neutralizing activity were incorporated at various sites on SLP. The successful expression of SLP chimeric immunodominant epitope 1 on the surface of *L.mucosae* G01 was confirmed through indirect immunofluorescence and transmission electron microscopy, revealing the formation of a transparent membrane. The findings demonstrate that the oral administration of *L.mucosae* G01, which expresses the SLP chimeric immunodominant gene epitope1, induces the production of secreted IgA in the intestine and feces of mice. Additionally, there is an elevation in IgG levels in the serum. Moreover, the levels of cytokines IL-2, IL-4, IFN-γ, and IL-17 are significantly increased compared to the negative control group. These results suggest that *L. mucosae* G01 has the ability to deliver exogenous antigens and elicit a specific mucosal immune response against PEDV. This investigation presents new possibilities for immunoprophylaxis against PEDV-induced diarrhea.

## Introduction

Porcine epidemic diarrhea (PED) is a profoundly contagious ailment that presents a considerable peril to piglet populations worldwide, resulting in substantial financial losses [1, 2]. The causative agent responsible for PED is the porcine epidemic diarrhea virus (PEDV), which exhibits a widespread prevalence porcine coronavirus and can induce severe and lethal infections in susceptible neonatal pigs [2]. The Spike (S) protein of PEDV plays a crucial role in the virus's entry into the host cell by mediating the interaction between the virus and the cell receptor, identified as the most immunogenic proteins [3, 4].

Vaccines are the most powerful tools available to prevent the spread of infectious diseases [5]. In the context of PEDV, where the initial point of infection resides in the oral and nasal mucosa, the significance of mucosal vaccines becomes paramount in thwarting the establishment, replication, and dissemination of the virus to other tissues through the local immune response [6, 7]. Mucosal vaccination presents numerous advantages compared to conventional vaccination techniques, such as simplified administration, decreased likelihood of allergic reactions, and cost reduction [8, 9]. Consequently, the development of a secure mucosal vaccine for PEDV that confers protection against both local and systemic infection is an imperative undertaking.

Nonetheless, a significant hurdle encountered with mucosal, oral, or nasal vaccinations is the potential denaturation of antigens within the gastrointestinal milieu, impeding their delivery to the intestinal mucosa and lymph nodes [10–12]. To surmount this challenge, the utilization of probiotic expression vectors has emerged as a viable approach for antigen delivery [8, 13]. Lactic acid bacteria (LAB) are Gram-positive bacterial agents that have gained considerable attention as adjuvants and carriers for augmenting the immunostimulatory effects of exogenous antigens on both cellular and systemic immune systems, specifically for the advancement of oral vaccines against enteroviruses [14]. In the meantime, the obstacle of attaining adequate levels of target protein expression persists as a noteworthy barrier. In order to tackle this concern, scholars have devised a surface display anchoring mechanism employing the structural domain of the S-layer protein (SLP) B subunit derived from *Lactobacillus acidophilus* (*L.acidophilus*) SLP, facilitated by the shuttle vector pELX1, which enables the exposure of said proteins on the surface layer of *Lactobacillus* [15]. This methodology has exhibited potential in enhancing the expression magnitude of desired proteins for application in vaccine formulation. Limited studies have been conducted on the potential of using SLP as a vector for surface display of heterologous proteins on SLP surface in Lactobacillus and Lactococcus lactis [16]. Recent research has investigated the resistance mechanisms of *L. acidophilus* Slp against various pathogens. Wang [17] conducted a study wherein the heterologous expression of Slp and pgsA by *Lactobacillus plantarum* NC8 resulted in improved resistance against gp43 and Nudix, thereby providing enhanced protection against Trichinella spiralis. Additionally, Arukha [18] demonstrated the efficacy of *L. acidophilus NCFM* SLP in alleviating inflammation associated with Inflammatory Bowel Disease. Furthermore, in their study, Uriza [19] utilized *L. acidophilus ATCC 4356* SLP to express the EITH7 antigen, resulting in enhanced resistance against *Escherichia coli* O157:H7. The findings of this research demonstrated the ability of the fusion protein to bind to the surfaces of different SLP-free *Lactobacillus* strains and *Lactococcus lactis*, while also exhibiting favorable immunogenic properties.

Bioinformatics is an interdisciplinary domain that concentrates on the organization, storage, and analysis of extensive volumes of data derived from biological experiments. It facilitates a comprehensive comprehension of biological functional mechanisms and molecular folding, rendering it an indispensable instrument for the advancement of vaccines [20–22]. However, despite its numerous advantages, the selection and design of protective immunogens against pathogens persist as a significant obstacle. Traditional laboratory techniques are inadequate in meeting the pressing requirements posed by contemporary pathogen outbreaks [21, 23]. Computer simulations have proven to be instrumental in addressing this challenge. By elucidating the crystal structure of PEDV S protein and gaining a deeper comprehension of the pathogen's invasion mechanism, bioinformatics analysis enables the prediction of viral attributes and identification of epitopes within the pathogen [24–26]. Consequently, the application of bioinformatics analysis in designing epitope peptide vaccines has demonstrated remarkable success in clinical settings. Jonas [27] developed CoVac-1, a vaccine designed to combat COVID-19, by tandemly connecting T cell epitopes and utilizing Toll-like receptor 1/2 agonist XS15 emulsification. This formulation has progressed to clinical trials. Similarly, Rodrigo Cañas-Arranz [28] engineered the B2T vaccine by expressing a B-cell epitope and linking it to a T-cell epitope. Notably, this vaccine exhibited complete protection in 70% of pigs challenged with the virus on day 25 after immunization. The conceptualization of these groundbreaking epitope vaccines represents a significant advancement in the field of disease management.

In this work, we manifested display of PEDV S protein B-cell linear epitope on *L. mucosae* G01 S-layer surface can induce a robust immunogenicity in mice. By utilized bioinformatics analysis, we predicate B-cell linear epitopes. We then employed *L. mucosa*e G01 as an expression vector to produce the SLP chimeric B-cell linear epitope 1. Then, the immunogenicity of the vaccine was assessed by oral administration of BALB/c mice. Our findings have significant implications for the development of oral PEDV vaccines and provide valuable insights for further research in SLP vaccines.

## Materials and methods

### Bacterial strain, virus, and plasmid

*Lactobacillus acidophilus* MG and *Lactobacillus mucosae* G01 (GenBank Accession: OR783178), plasmid-free strains obtained through laboratory isolation and preservation, were cultured in MRS medium (Guangdong Huankai Biological). Vero cells were cultured using DMEM medium (Gibco™, Grand Island, NY, USA; 119,955), and fetal bovine serum (FBS) was obtained from Gibco™ (10,099). The plasmid pTRK892 was acquired from Addgene (http://www.addgene.org). The PEDV-HZ strain was sourced from the Institute of Animal Health, Guangdong Academy of Agricultural Sciences (GenBank Accession: OP191700.1).

### B-cell epitope prediction

Immunoinformatics tools, specifically the ABCpred server [29] (https://webs.iiitd.edu.in/raghava/abcpred/ABC_submission.html) with a threshold of 0.75 and BepiPred 2.0 [30] (http://www.cbs.dtu.dk/services/BepiPred/) with a threshold of 0.5, were utilized to predict B-cell epitopes. The antigenicity of the identified epitopes was evaluated using the Vaxijen 2.0 server [31] (http://www.ddgpharmfac.net/vaxijen/VaxiJen/VaxiJen.html). Furthermore, the screening process included consideration of hypoallergenicity (http://www.ddg-pharmfac.net/AllerTOP/) [32], low toxicity [33] (http://crdd.osdd.net/raghava/toxinpred/), and good water solubility (http://crdd.osdd.net/raghava/toxinpred/) when selecting peptides. The Alphafold 2.0 online platform [34] (https://colab.research.google.com/github/sokrypton/ColabFold/blob/main/AlphaFold2.ipynb) was utilized for conducting structural analysis on the S protein monomers, employing the template (PDB: 7w6m). Subsequently, the identified peptides underwent additional characterization through the utilization of Pymol software [35].

### Peptide synthesis and preparation

The peptides corresponding to the predicted epitopes were synthesized by Shanghai Bioengineering Co., Ltd., China. A total of seven peptides, namely SQEPFDPSGY, ASTNFVDAL, TISEEALQL, YLALQTDVL, TLGPTANNDVTT, SAGEDGISYQP, and GPRLQPY (the latter serving as a reported positive control), were successfully synthesized with a purity exceeding 95%. To enhance the immune response to peptides, Keyhole Limpet Hemocyanin (KLH) carrier protein was conjugated to the C-termini of the synthesized peptides. Cys amino acid residues were added to the synthetic peptide's C-terminus and an SMCC coupler was used to facilitate KLH coupling. Subsequently, these peptides were dissolved in sterile distilled water at a concentration of 1 mg/mL.

### Gene amplification and construction of expression plasmids

The immunodominant epitope 1 of the chimeric PEDV S protein, which incorporates the Amp resistance gene fusion SLP target protein, was integrated into the original pTRK892 plasmid. The Amp resistance gene was obtained from pMD19T using specific primers AmpF: cg*GCGGCCGC*ctggtaccgacagttaccaatgcttaatcagtga and AmpR: gc*GTCGAC*caggtggcacttttcggggaaatgtg, where *Sal I* and *NotI* cleavage sites are indicated by underlining. SLP-specific amplification primers were SLPF: ggc*GAATTC*atgaagaaaaattt and SLPR: ggtacc*GCGGCCGC*ttatctaaagtttgcaacctt, with *EcoR I* and *Not I* restriction endonuclease sites also indicated by underlining. The pre-optimization sequence of epitope 1 was TCGCAAGAGCCTTTTGACCCTAGTGGTTAC, and the post-optimization sequence was AGTCAAGAACCATTTGATCCATCAGGTTAC. Erythromycin (Sigma) was administered at a concentration of 10 μg/mL, while ampicillin and penicillin were maintained at 100 μg/mL.

### *L. mucosae* G01 competent cells preparation and transformation

The preparation protocol was adapted from a previously described procedure [36]. Overnight cultures of *L. mucosae* G01 were expanded to a volume of 200 mL, and when the optical density (OD) reached 0.5, the bacteria were subjected to centrifugation at a speed of 8000×*g* for a duration of 20 min. Subsequently, the resulting pellet was washed three times. Competent cells were prepared by resuspending them in a 10% glycerol solution containing 1 mol/L sucrose, with a volume of 10 mL. The cells were then divided for preservation. Subsequently, the competent cells were placed in a pre-cooled electrotransfer cup, and 0.5 μg of the expression plasmid was added to 50 μL of competent cells for thorough mixing. The resulting mixture was kept on ice for a duration of 30 min. For electrotransformation into the competent cells, a voltage of 2.00 kV was applied using a 2.5 ms single-pulse in a 0.1-cm electrotransfer cup. The transformed cells were subsequently introduced into MRS medium containing a concentration of 1 mol/L sucrose and cultured at a temperature of 37 °C for a duration of 5 h. Following this, the cells underwent centrifugation and were then plated on erythromycin MRS plates that were supplemented with a concentration of 10 μg/mL of erythromycin. The plates were incubated at a temperature of 37 °C for a period of 30–48 h. Positive colonies were identified as *L. mucosae* G01-pTRK892-Amp-Slp-epitope 1. White positive single colonies were cultivated, and identification was carried out using the primers SLP-IF: AGAACCATTTGATCCATCAGGTTACGGTGGTGGTGGTAGCGCTACTACTATTAACGCAA; and SLP-IR: GGCCGCTTATCTAAAGTTTGC; 892F: GTTGAAGAAGCTAAGAAGGCT; and T1R: GTAACCTGATGGATCAAATGGTTCTTGACT in accordance with established protocols.

### SDS-PAGE and western blot

Single colonies of *L. mucosae* G01-pTRK892-Amp-Slp-epitope 1 were accurately identified and subsequently cultured in 1 mL of MRS medium supplemented with 10 μg/mL erythromycin for a duration of 15 h. The colonies were then subjected to centrifugation at a speed of 8000×*g* for a period of 5 min, followed by three rinses utilizing PBS. To induce lysis, bacterial lysate (Omega Bio-TEK, L10TG) was introduced, and the resulting lysate was combined with 5 × upsampling buffer and heated for 10 min through boiling. Subsequently, sodium dodecyl sulfate–polyacrylamide gel electrophoresis (SDS-PAGE) and western blot analysis were conducted.

The aforementioned samples underwent separation using a 12% SDS-PAGE technique, were subsequently transferred onto a PVDF membrane, and were blocked at ambient temperature utilizing a 5% skimmed milk solution for a duration of 2 h. Sera derived from KLH-epitope 1 immunized mice were then subjected to incubation at a dilution of 1:500, also at room temperature, for a period of 1 h. Following this, the samples were washed thrice with PBST and subsequently incubated with HRP-goat anti-mouse IgG (H + L) at a dilution of 1:5000 for 1 h at room temperature. This was followed by an additional three washes with PBST. Finally, color development was achieved through the utilization of an ECL reagent.

### Indirect immunofluorescence detection of *L. mucosae* G01 surfaces display SLP chimeric immunodominant peptides

The recombinant bacteria, *L.mucosae* G01-pTRK892-Amp-Slp-epitope 1, were cultivated in MRS medium for a duration of 12 h. Following this, the bacteria underwent triple washes with PBST and were centrifuged at a speed of 8000×*g* for a period of 5 min. The bacteria were then affixed to slides and subjected to overnight fixation at a temperature of 4 °C using a 4% paraformaldehyde solution. After three subsequent washes with PBST, the samples were sealed with a 5% skimmed milk solution for a duration of 2 h. Immunofluorescence staining was performed by incubating the samples with polypeptide-immunized mouse serum at a dilution ratio of 1:200. The incubation occurred at a temperature of 4 °C for the duration of one night. Subsequently, the samples underwent three washes with PBST solution. Afterward, the samples were exposed to Alexa Fluor 594-conjugated goat anti-mouse IgG (H + L) fluorescent secondary antibodies (red) for a duration of 1 h. Following this, the specimens were subjected to three additional washes with PBST solution. Finally, the samples were mounted onto slides and covered with coverslips in preparation for observation using fluorescence microscopy.

### Transmission electron microscopy of recombinant *L. mucosae* G01 surface displaying SLP chimeric immunopeptide

The method described by Garrote [37] was utilized with minor modifications. The recombinant bacterium *L. mucosae* G01-pTRK892-Amp-Slp-epitope 1 was diluted with *L. acidophilus* MG and *L. mucosae* G01 in PBS, followed by centrifugation at 8000×*g* for 5 min. The resulting cells were then stained with 2% Phosphotungstic Acid (PTA, Sigma, USA) for a precise duration of 2 min. Subsequently, the combined organisms were applied to a 150-mesh Formvar grid and carefully examined using a HITACHI transmission electron microscope HT7700 operating at 90 kV.

### Mouse immunization

Water-soluble peptides were thoroughly emulsified with an equal volume of complete Freund's adjuvant (Sigma, SLCH2657) in accordance with a comprehensive experimental design. Negative controls were established using KLH, resulting in a total of 8 groups, each consisting of 5 mice. Subsequently, this emulsion, containing 0.2 mg of each peptide, was subcutaneously injected into female BALB/c mice. Booster immunizations were then administered at two-week intervals, utilizing incomplete Freund's adjuvant. Blood samples were collected at intervals of 14 days, 28 days, and 42 days from the orbital area to isolate serum.

Recombinant bacteria, namely *L.mucosae* G01-pTRK-892, *L.mucosae* G01-pTRK-Slp-892, *L.mucosae* G01-pTRK892-Amp-Slp-epitope 1, and *L.mucosae* G01, were subjected to a single wash with sterile PBS. The concentration of the bacterial solution was carefully adjusted to OD450 = 1.50 ± 0.05, equivalent to approximately 10^10^ CFU/mL, using a spectrophotometer. Following this, mice were orally administered the bacteria for three consecutive days, with a two-week interval between each administration (days 14–16, 28–30, and 42–44). Fecal samples were systematically collected on days 0 and 56. Fresh feces from three randomly selected mice in each group were collected and added to a solution of PBS containing 1 mmol/L PMSF and 1% bovine serum albumin (BSA) with great care. Additionally, at 17, 30, and 45 days after immunization, three mice from the immunized group were euthanized and subjected to a meticulous procedure in which 0.5 mL of intestinal surface mucus was carefully scraped from their small intestines after flushing with sterile PBS buffer. The resulting mixture was then centrifuged at 3500×*g* for 5 min to separate the supernatant, which was subsequently stored at -80 °C for further analysis.

### Enzyme-linked immunosorbent assay (ELISA) for intestinal and feces IgA and serum IgG

Epitope-specific peptides, conjugated with keyhole limpet hemocyanin (KLH), were solubilized in a bicarbonate solution at a pH of 9.6, resulting in a concentration of 5 ng. Elisa plates were then coated with this peptide solution and incubated overnight at a temperature of 4 °C. Subsequently, the plates were sealed with PBS containing 2% BSA for a duration of 2 h at a temperature of 37 °C. Following this, the plates were subjected to an incubation period of 1 h at a temperature of 37 °C with 100 μL of serum samples, feces fragmentation suspension, and intestinal lavage, all diluted at a ratio of 1:100. After the incubation period, the plates were subjected to three washes with PBST. Following this, each well was treated with either HRP-conjugated goat anti-mouse IgG (1:20,000) or goat anti-mouse IgA secondary antibody (Invitrogen, XL3790084A) (1:500), which was coupled to HRP, and incubated at 37 °C for 1 h. Subsequently, the plates were washed three more times with PBST. The color development was initiated by adding TMB substrate, and the absorbance was measured at a wavelength of 450 nm.

### Cytokine detection

The analysis of IFN-γ, IL-2, IL-4, and IL-17 cytokines in serum was conducted using a commercially procured Cytokine Assay Kit from R&D.

### PEDV neutralisation assay

Neutralization assays were performed using serum from mice immunized with peptides, following the methodology described by Maier [38]. Briefly, the serum was incubated at 56 °C for 30 min. Subsequently, 50 μl of each sample was meticulously prepared in a 96-well plate, following a third-fold serial dilution (ranging from 1:3 to 1:81). The diluted serum was then combined with 50 μl of a diluent containing 200TCID_50_ PEDV (10% concentrated Hanks balanced salt solution and 0.1% BSA). Vero cells, which were employed for viral infection, were subsequently exposed to the antibody-virus mixture. The entire experimental setup was subjected to incubation in a 5% CO_2_ incubator at a temperature of 37 °C for a duration of 3 days. The virulence of the virus was assessed based on the cytopathic effect, and the results were quantified using the Reed-Muench method.

### Statistical analysis

All collected data were subjected to analysis using GraphPad Prism software v8.0 (GraphPad Software Inc., San Diego, CA, USA). The outcomes were presented as the mean ± SEM of a minimum of three independent experiments. One-way analysis of variance, followed by the Tukey–Kramer post-test, was employed for statistical analysis. A *p*-value less than 0.05 was considered to indicate statistical significance.

## Results

### Bioinformatics analysis and identification for epitope-based vaccine design against PEDV

**Fig. 1:**
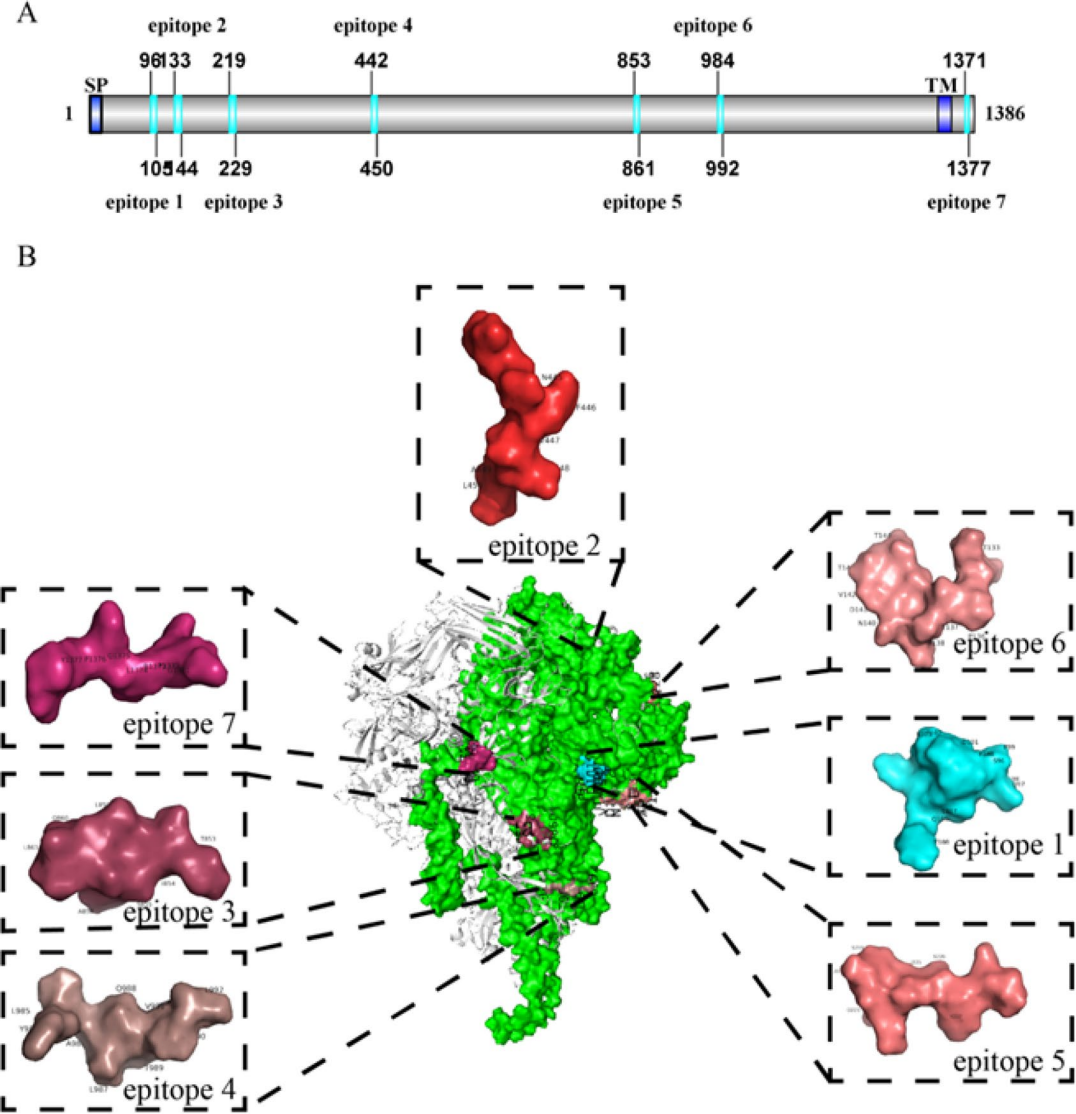
Immunodominant epitopes of PEDV S protein screened by bioinformatics. **A** The primary structure of the PEDV S protein was analyzed, and the precise location of the synthetic immunodominant epitopes within the S protein was elucidated using the IBS v1.0 software. The functional structural region of the S protein is visually represented in a dark blue color, with the signal peptide (SP) spanning positions 1–19 and the transmembrane region (TM) structural domain from 1328 to 1350. **B** The specific peptide positions of interest are highlighted in a bright blue color. In addition, immunodominant epitopes were identified using the AlphaFold v2.0 online site prediction tool and then visualized on the protein surface using Pymol software. The trimeric structure of the S protein is depicted in a silver-gray color, while the monomeric S structure is shown in green. Distinct colors were assigned to characterize immunodominant epitopes 1–7

To analyze the immunodominant linear epitopes of the PEDV S protein using a combination of bioinformatics and immunoinformatics predictions, we successfully identified 12 distinct peptides through the ABCpred server (with a threshold of 0.75) (data not shown). Additionally, the BepiPred-2.0 server, set at a threshold of 0.5, provided predictions for 24 contiguous B-cell epitopes (data not shown). Following that, we conducted an evaluation of antigenicity using the Vaxijen 2.0 server, wherein we identified peptides with high scores as potential B-cell linear epitopes. Our selection criteria were rigorous, emphasizing peptides that exhibited low allergenicity, low toxicity, and favorable water solubility, as evidenced in Table 1. The screened peptides were predominantly situated in the N-terminus of the S protein, with the intracellular region containing epitope 7 serving as a positive control (Fig. 1A). In the process of establishing a structural framework for the S protein, we employed the resolved PEDV S protein crystals (PDB: 7w6m) as a reference to simulate the S protein monomer using Alphafold 2.0 software. Through comparative analysis between the simulated proteins and the actual crystals, we observed an RMSD value of 2.918 Å, thereby confirming the viability of generating a precise sequence model for the virus strain through computational means. To visualize the predicted B-cell linear epitopes, we utilized Pymol software to present them in a surface format. The precise localization of amino acids on screened peptides was determined by comparing residues in the protein structure. It is noteworthy that the selected peptides demonstrated surface accessibility within S protein monomers, as depicted in Fig. 1B.

**Table 1:** Comprehensive screening

No.	Sequence	Allergic	ToxinPred	Hydrophobicity	Charge
1	SQEPFDPSGY	No	No	− 0.19	− 2.00
2	ASTNFVDAL	No	No	0.04	− 1.00
3	TISEEALQL	No	No	− 0.03	− 2.00
4	YLALQTDVL	Yes	No	0.09	− 1.00
5	TLGPTANNDVTT	No	No	− 0.10	− 1.00
6	SAGEDGISYQP	No	No	− 0.12	− 2.00
7	GPRLQPY	No	No	− 0.36	2.00

### Identification of immunodominant peptides

**Fig. 2:**
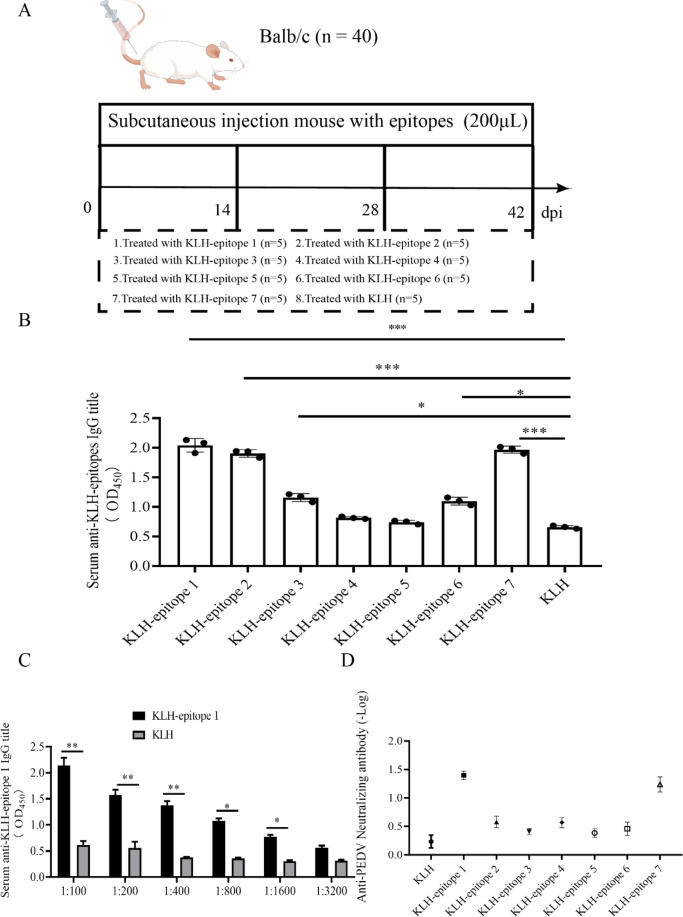
Induction of in vivo immune responses in mice immunised with synthesised immunodominant peptides. **A** Strategies for synthesising immunodominant peptides using Figdraw. **B** Detection of IgG title in serum of mice on day 56. **C** immune epitope 1 serum titre assay. **D** Peptide immunisation with murine serum neutralisation test. The results, presented as the mean ± SEM of three independent experiments, indicate no significant difference denoted by "ns". However, statistically significant differences between groups are denoted by *p < 0.05, **p < 0.01, or ***p < 0.001

To assess the effectiveness of the synthetic peptides, seven peptides were synthesized and conjugated to the carrier protein KLH to enhance immunoreactivity, as illustrated in Fig. 2A. Subsequently, the measurement of serum antibody levels via ELISA yielded compelling evidence. Our results indicate that the synthesized peptides (epitope 1, 3, 6, and 7) induced strong immune responses in mice compared to the untreated group (*p* < 0.05) (Fig. 2B). Specifically, the OD_450_ value of the epitope 1 peptide serum was 2.083 (*p* < 0.001), while the epitope 2 peptide serum had a value of 1.873 (*p* < 0.001) compared to the untreated group. At a serum dilution of 1:1600, KLH-epitope 1 showed a significant difference in serum IgG levels compared to the KLH control. Based on these findings, the specific serum anti-KLH-epitope 1 titer was determined to be 1:1600 (Fig. 2C). Additionally, when the immunizing peptide serum was combined with 200 TCID_50_ PEDV for Vero cells, the neutralizing antibodies anti-epitope 1 group was significantly higher (Fig. 2D). These findings underscore the capability of the screened epitope 1 peptide to induce robust neutralizing antibodies against PEDV.

### Recombinant *L. mucosae* G01 expression strategy for SLP Chimeric Epitope1

**Fig. 3:**
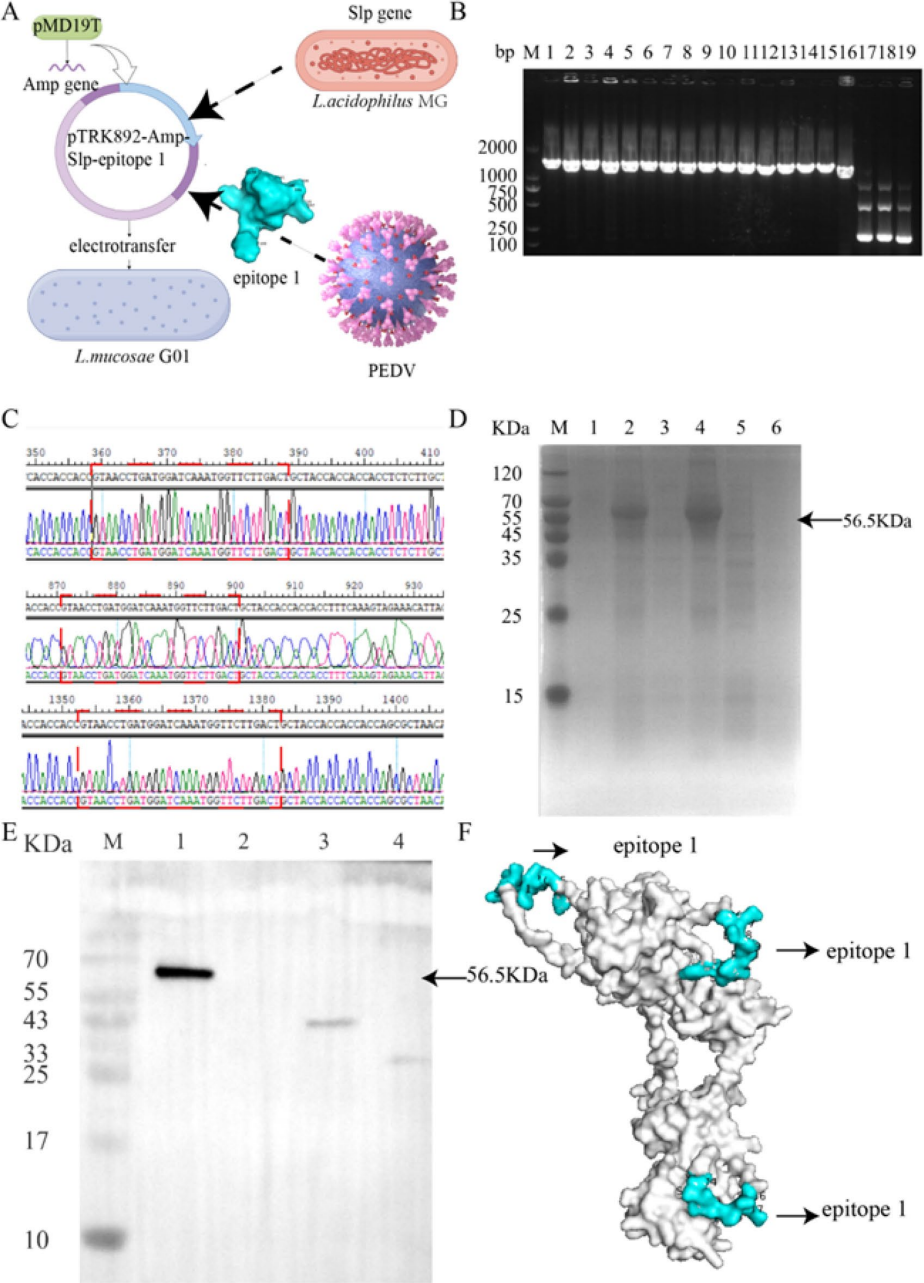
Strategy and expression of epitope 1, an immunodominant epitope of *L. mucosae* G01 chimeric PEDV S protein. **A** A schematic illustration of *L.mucosae* G01 expressing SLP chimeric epitope 1 was created using the Figdraw online website. **B** The resulting PCR products were subsequently separated using 1.2% agarose nucleic acid gel electrophoresis. The DL2000 Marker was used as a reference, and lanes 1–16 were designated for testing SLP fragments with an expected band size of 1684 bp. Lanes 17–19 were specifically designated for the evaluation of epitope1 presence, with anticipated band sizes of 138 bp, 615 bp, and 1125 bp. **C** Comparative analysis revealed the presence of the optimized epitope1 gene, with the identified target fragments highlighted in red boxes. **D** The expression of *L. mucosae* G01-pTRK892-SLP-epitope 1 was detected by SDS-page. The lysate (lane 2) and supernatant (lane 1) of *L. mucosae* G01 expressing the SLP chimeric immunoepitope 1 exhibited the presence of a 56.5 kDa chimeric protein. Additional lanes on the gel included *L. acidophilus* MG supernatant (lane 3), *L. acidophilus* MG lysate (lane 4), *L. mucosae* G01 (lane 5), and a blank control (lane 6). Arrowheads were employed to highlight the target bands in the gel. **E** Western blot analysis was conducted to detect the presence of recombinant *L. mucosae* G01 expressing SLP chimeric immunodominant epitope 1. Lane 1 represented recombinant *L. mucosae* G01 expressing SLP chimeric epitope 1, while lane 2 represented recombinant *L. mucosae* G01 expressing SLP. Lane 3 represented recombinant *L. mucosae* G01, and lane 4 represented *L. acidophilus* MG. Arrows were utilized to highlight the target bands on the membrane. The protein marker was denoted as M. **F** The protein structure of the epitope 1 chimeric SLP was computationally modeled using AlphaFold 2.0. The structural representation highlights the epitope 1 structure in blue and the SLP structure in gray. Arrows are utilized to indicate the precise location of epitope 1 within the SLP structure

In order to optimize our screening procedure, we integrated an ampicillin resistance gene into the pTRK892 vector. The desired protein, SLP, derived from *L. acidophilus* MG, was amplified and cloned into the target fragment. To achieve the fusion of immunodominant epitopes, codons were optimized using online bioinformatics tools. The epitopes were successfully incorporated into the SLP protein through homologous recombination, with the strategically connected epitopes using the flexible linker *GGGGS* (Fig. 3A). Following electrotransformation, monoclonal bacteria were selected for PCR identification, confirming the presence of the target gene (Fig. 3B). The sequencing results aligned with our anticipated outcomes (Fig. 3C), demonstrating that the immunodominant epitopes were fused into the SLP protein in three distinct segments. Subsequently, SDS-PAGE and Western blotting analyses were conducted on separate colonies. A distinct band with a molecular weight of 56.5 kDa was observed, as depicted in Fig. 3D and E. Following the incorporation of the immunodominant epitope 1 into SLP, structural simulations were conducted, as illustrated in Fig. 3F.

### Surface demonstration of recombinant *L. mucosae* G01 Expressing SLP Chimeric Immunodominant Epitope 1

**Fig. 4:**
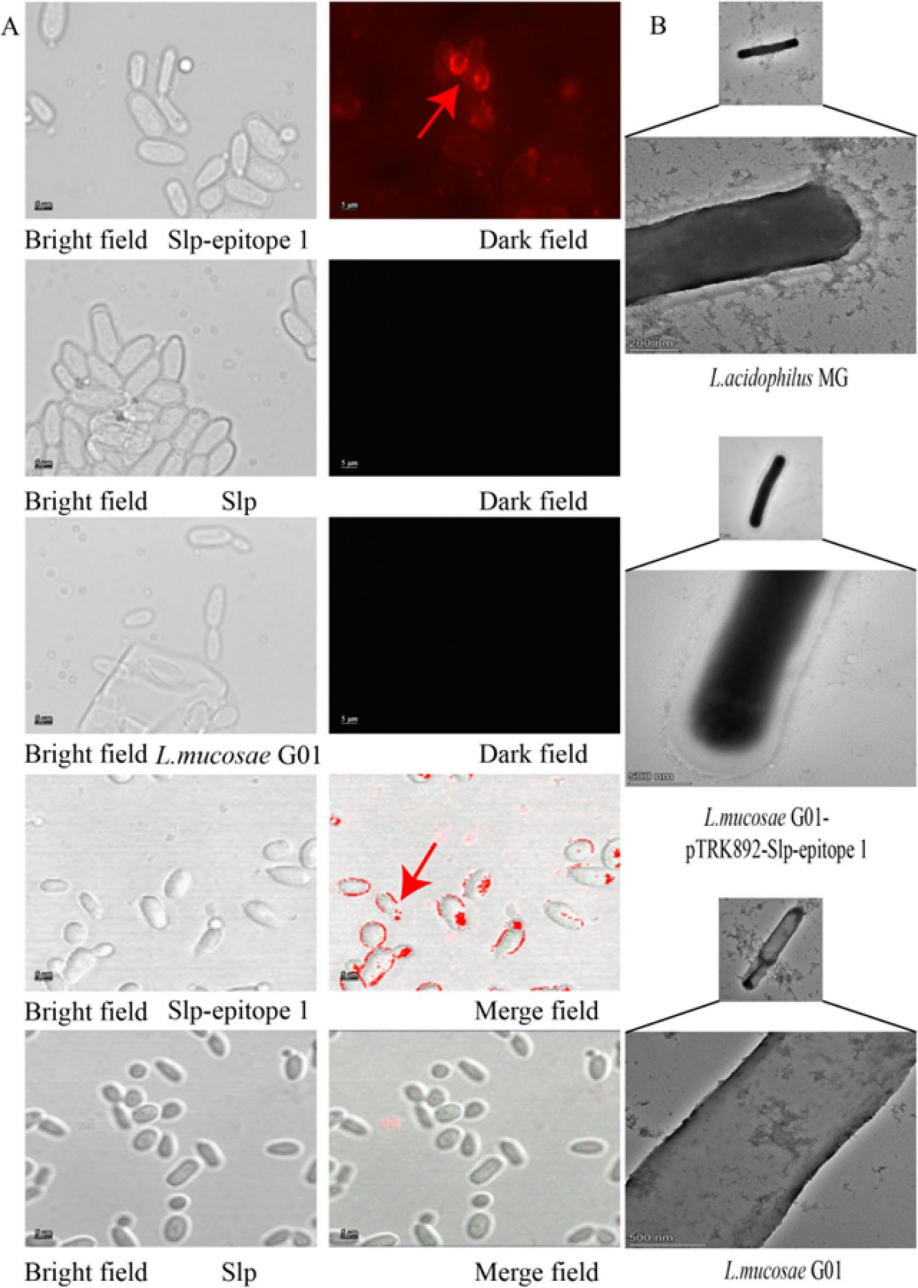
Recombinant *L. mucosae* G01 SLP surface display. **A** The expression of the SLP chimeric epitope 1 in recombinant *L. mucosae* G01 was examined using a Zeiss 710 laser confocal microscope. Incubation was carried out with *L. mucosae* G01-pTRK892-Slp-epitope 1 serum, employing Alexa Fluor 594-conjugated goat anti-rabbit IgG antibody (red) as the secondary antibody. **B** The localization of epitope 1 to bacterial membranes was successfully demonstrated through the use of HITACHI transmission electron microscopy in *L. mucosae* G01 fusions expressing SLP chimeric epitope 1, as indicated by the arrowheads

In our study, the indirect immunofluorescence results demonstrated the effective surface expression of SLP and SLP chimeric epitope1 by *L. mucosae* G01 (Fig. 4A). Notably, the fluorescence signals indicated a prominent display of both entities on the bacterial surface. Furthermore, our observations using transmission electron microscopy provided additional confirmation (Fig. 4B). Specifically, the fusion of SLP chimeric epitope 1 by *L. acidophilus* MG and *L. mucosae* G01 resulted in the presence of a distinct transparent membrane. In contrast, the native state of *L. mucosae* G01 did not possess the aforementioned associated protein structure. These findings indicate that exogenous SLP can be successfully directed to the membrane surface of *L. mucosae* G01, thereby confirming its effectiveness as a potent means of membrane display.

### Recombinant *L. mucosae* G01 enhances immune function in mice

**Fig. 5:**
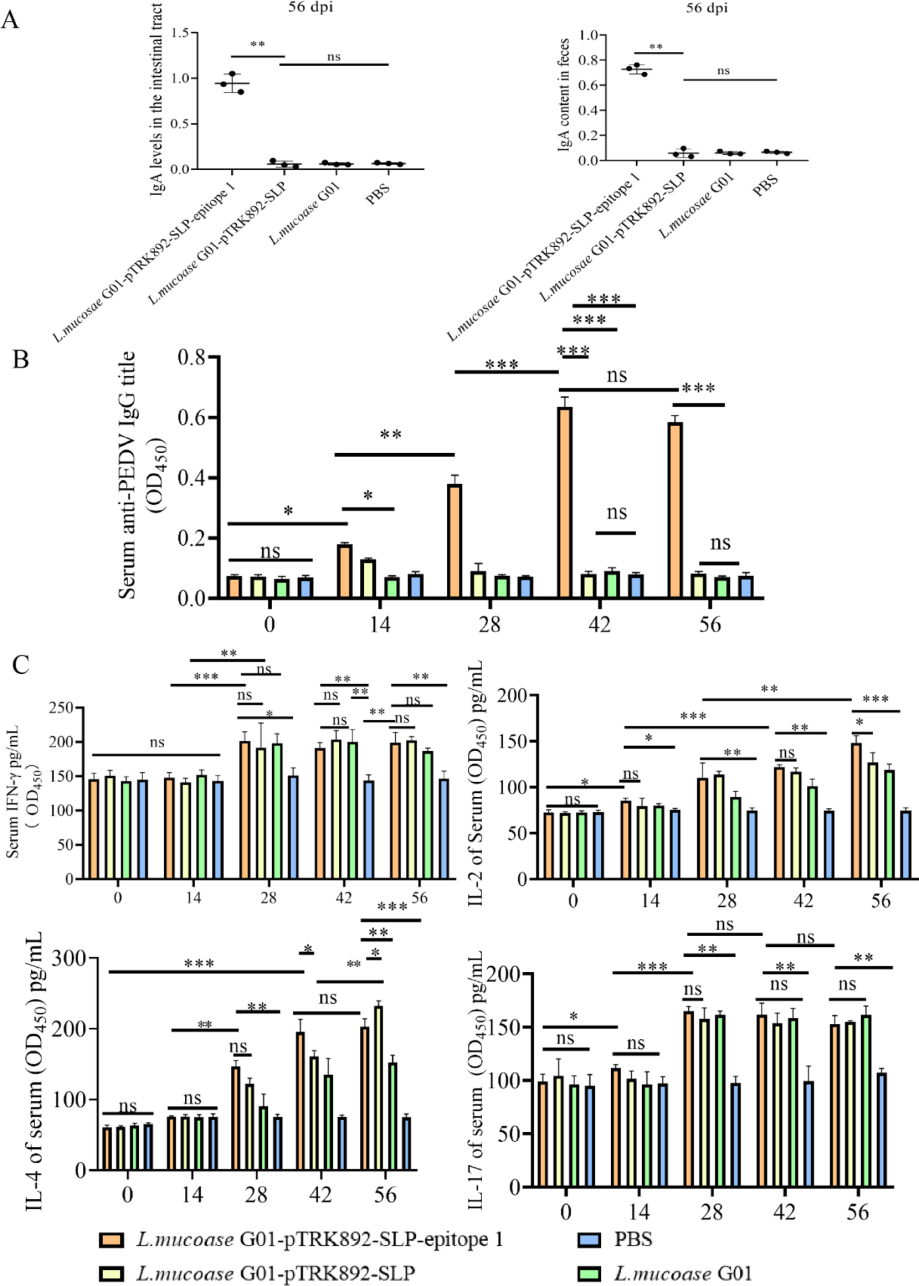
*L. mucosae* G01-pTRK892-SLP-epitope 1 induce immune responses in mice. **A** Elisa assay for IgA in gut and faeces. **B** Evaluation of changes in IgG levels in the serum of mice orally administered *L. mucosae* G01*-*pTRK892-SLP-epitope 1*.* **C** Serum cytokine levels were measured in accordance with the assay instructions provided by the R&D Commercial Kit. The results are presented as the mean ± SEM of three independent experiments. The abbreviation "ns" denotes no significant difference. Statistically significant differences between groups are denoted by *p < 0.05, **p < 0.01, or ***p < 0.001

To evaluate the immunogenicity of our constructs, we administered *L. mucosae* G01 containing the chimeric immunodominant epitope 1 to mice. Our results reveal a substantial production of specific sIgA antibodies in both intestinal and fecal samples (Fig. 5A). Notably, the concentration of IgA antibodies in the gut was significantly higher than that in the faeces, providing evidence for the validity of our immunisation method. Additionally, serum derived from mice immunized with *L. mucosae* G01-pTRK892-Amp-Slp-epitope 1 demonstrated the production of IgG antibodies specifically targeting anti-PEDV, as indicated by an OD_450_ value of 0.634 (Fig. 5B). This observation suggests that our genetically engineered bacteria stimulated a systemic immune response. The utilization of cytokine kits enabled the identification of cellular immune responses in mice. Notably, heightened concentrations of IL-2, IL-4, IFN-γ, and IL-17 were detected, signifying the initiation of a comprehensive immune response that encompasses both Th2 and Th1/Th17 cellular immunity (Fig. 5C). These findings collectively emphasize the diverse immunological influence of our immunization approach.

## Discussion

Vaccination has emerged as a prevalent prophylactic strategy against PEDV, with the utilization of both live attenuated and inactivated vaccines. However, the primary challenge with the two existing vaccines lies in their relatively limited efficacy in conferring immune protection [39–44]. Given the ineffectiveness of conventional vaccines in conferring mucosal immune protection against the enterovirus PEDV, we endeavored to leverage probiotics as vectors to express immunodominant epitopes, aiming to provide robust protection against PEDV infection. In the trials conducted to assess the protection of piglets against PEDV, a significant positive correlation was observed between the levels of IgA and IgG antibodies in both the intestinal-associated lymphoid tissues and blood of the piglets [45], as well as the titers of IgG and IgA antibodies in their serum [45]. Despite the existence of a single serotype of PEDV, the cross-protective effect between variants of PEDV with distinct genomes was found to be incomplete [46]. Additionally, sows that had been previously infected with PEDV demonstrated the ability to generate lactation immunity, effectively safeguarding lactating piglets from morbidity [47].

Hence, the development of a highly efficient oral vaccine for PEDV is of utmost importance. In this regard, the utilization of bioinformatics simulation to predict immunodominant epitope targets provides a more precise and expedited alternative to conventional phage surface display screening methods [48, 49]. In our experimental study, we successfully identified the immunodominant epitope ^96^SQEPFDPSGY^105^ through bioinformatics screening and subsequent chemical synthesis. Notably, the serum derived from immunizations with this epitope exhibited a neutralizing activity of 1:32 after four rounds of immunizations. Despite the limited immune response activation observed in vivo, this peptide molecule exhibited superior efficacy in neutralizing PEDV compared to the ^1368^GPRLQPY^1374^ (2C10) peptides. The ELISA assay conducted on the immunodominant epitope peptide also demonstrated strong binding activity, with a serum dilution potency of 1:1600. Kanokporn Polyiam et al. [22] successfully employed various online software tools, including BepiPred-2.0, Emini, Kolaskar & Tongaonkar, Parke, and Emini, to screen for B-cell linear epitopes, ultimately identifying ^88^EIGISQEPFDPSGY^101^. The neutralizing activity of the peptide was confirmed indirectly by conducting chemical synthesis epitope and viral competition experiments using PEDV-positive pig sera. This led to the complete neutralization of CV777 (G1a) infection. Yu Ruisong et al. [50] employed a similar approach to screen ^91^ISQEPFDPSG^100^ and observed that the expressed fragment, which was tagged with GST through the *Escherichia coli* expression system, induced a specific immune response against the SD2014 (G2b) strain. These investigations provided conclusive evidence of the robust immune activity exhibited by the immunodominant epitope. To ensure the accuracy of the immunodominant epitopes, protein structure simulations were performed using AlphaFold 2.0, with PDBID: 7w6m serving as a template. The immunodominant epitopes ^88^EIGISQEPFDPSGY^101^ and ^91^ISQEPFDPSG^100^ were subjected to a comparative analysis using Pymol visualization software. Notably, the structural component ^96^SQEPFDP^102^ was found to be prominently located on the surface of the S protein, in contrast to its positioning in ^88^EIGISQEPFDPSGY^101^, where ^88^EIGI^91^ is inwardly recessed. Comparative analysis of the sequences of 59 PEDV strains indicated that the immunodominant epitope 1 exhibited a significant level of conservation across various strain types, including G1a, G1b, G2a, and G2b. Ongoing investigations are currently focused on identifying neutralizing epitopes encompassing ^1368^GPRLQPY^1374^ (2C10) [48], S1 (residues 1–219), S1^B^ (residues 510–640) [51], P4B-1 (residues 575–639), E10 (residues 435–485) [52], SS2 (residues 748–755), and SS6 (residues 764–771) [49]. In comparison to the aforementioned peptides, the epitope 1 sequence, ^96^SQEPFDPSGY^105^, demonstrates a relatively concise nature, precision, and remarkable efficacy in neutralization. These attributes render it a highly auspicious candidate for the development of diagnostic and therapeutic agents.

The *L. acidophilu*s SLP demonstrates the ability to self-assemble into uniformly crystallized planar structures and can non-covalently attach exogenous proteins to the bacterial wall [53–55]. Additionally, SLPs show promising potential as carrier adjuvants, in nanoscale materials, and for biomimetic applications [56–58]. Notably, transmission electron microscopy revealed a significant presence of filamentous proteins surrounding the surface of *L. acidophilus* MG and the surface of *L. mucosae* G01 fusion expression of the SLP chimeric immunodominant epitope 1, while *L. mucosae* G01 itself lacks this protein. This phenomenon may be attributed to the electrostatic impact generated by the non-covalent bonding of SLP protein, which has the ability to adsorb secretions surrounding lactobacillus. SLP proteins have been demonstrated efficacy as carriers for expressing exogenous proteins. In our experimental trials, SLP proteins served as the anchoring protein to specifically target three identical immunodominant peptides to the surface of the lactobacillus membrane. To prevent any disruption to the helical structure and β-folding of SLP proteins, insertion sites for the immunodominant peptides were strategically chosen within highly hydrophilic amino acid regions, specifically at positions 31, 189, and 309. In comparison to alternative surface-display techniques, namely psaA, pspA [59, 60], and pgsa [61], SLP surface display demonstrated notably enhanced expression levels without the requirement of supplementary inducers. Available reports suggest that SLP surface display can account for a significant proportion, approximately 10–15%, of the overall bacterial proteins, which is a substantial quantity for *Lactobacillus* [62]. This heightened expression level confers a distinctive advantage to lactobacillus in the expression of exogenous proteins. Leveraging the flexibility and adhesive properties of SLP, we employed this method to express the trimerization-dominant epitope structure of the PEDV S protein. This approach yielded improvements in both overall protein expression and the duration of protein hydrolysis. As a result, the protein was able to sustain immune response stimulation on the bacterial surface for an extended period of time. In recent years, there has been a growing interest in studying the antiviral properties of the SLP protein itself. For example, Zhang [63] discovered that the *L. acidophilus* SLP protein could counteract PEDV-induced apoptosis in Vero cells by interacting with apoptotic factors caspase-8 and caspase-3. Additionally, Prado Acosta M [64] utilized SLP to interact with DC–SIGN, effectively blocking SFV infection. In our study, the strain *L. mucosae* G01, which was obtained from the fecal samples of Bama pigs, exhibited significant inhibition against PEDV. We utilized the fusion expression of the SLP by *L. mucosae* G01 as a foundational framework for the heterologous expression of immunodominant epitopes that specifically target pathogens. This strategy incorporates multiple stacking effects to effectively counteract pathogen invasion.

To a certain extent, the surface display of SLP chimeric immunodominant epitope 1 on *L. mucosae* G01 induced robust mucosal, humoral, and cellular immune responses to a certain extent. IgA, primarily present on mucosal surfaces, plays a critical role in defending against respiratory and digestive tracts, among other mucosal surfaces [43, 65]. Therefore, the assessment of IgA levels emerged as a crucial determinant of resistance to porcine epidemic diarrhea virus (PEDV) in piglets. Notably, specific IgA antibodies were identified in the intestinal tract and fecal matter, with higher concentrations of IgA observed in the intestinal tract compared to the feces. This disparity could potentially be attributed to the colonization of *L. mucosae* G01 in the intestinal tract following perigastric passage, which stimulates the differentiation of intestinal B-lymphocytes. Furthermore, evidence suggests that *L. acidophilus* NCFM SLP facilitates the maturation of immature dendritic cells and Th cells, as demonstrated by Konstantinov [66]. Unfortunately, we have observed low levels of IgA antibodies in the serum, potentially attributable to the autonomous functioning of the mucosal immune system separate from the systemic immune system. Mucosa-associated lymphoid tissues exhibit selective utilization of effector mechanisms and adjust their potency to hinder compensatory mechanisms of immune depletion when stimulated by a wider array of antigens [67, 68]. In order to evaluate the humoral immune response elicited by the lactobacillus-vectored vaccine, we have detected heightened levels of serum IgG antibodies specific to PEDV in mice, reaching their maximum after six weeks. Mucosal defense exhibits a notable superiority over the peripheral immune system in its efficacy in preventing infections. Our observations, in line with the findings of Li Yi, In-Chan Hwang, and other researchers [69–71], indicate comparable or slightly diminished levels of IgG, which may be linked to the length of our encapsulated peptide. The activation of various structural subunits within the intact S protein antigen may potentially induce undesirable inflammatory responses. Optimal vaccine vectors should selectively target PEDV-specific receptors or encompass combination vaccines comprising multiple epitope preparations. In our experimental study, both SLP and SLP chimeric immunodominant epitope 1 elicited a rise in cytokine levels, encompassing IFN-γ, IL-2, IL-4, and IL17. IFN-γ and IL-2, predominantly generated by Th1 cells, may collaborate with IFN-γ to augment cellular immune responses. IL-4, primarily synthesized by Th2 cells, governs humoral immune responses by fostering the expansion of activated B-cell and impeding the activation and cytokine release by Th1 cells. The cytokine IL-17, which is secreted by Th17 cells, primarily functions to enhance the activation of neutrophils and macrophages, thereby facilitating the release of inflammatory mediators and participating in tissue repair [72–74]. Our findings demonstrate that both *L. mucosae* G01 fusion expressing SLP and expressing SLP chimeric immunodominant epitope 1 exhibit a similar tendency towards elevation. Furthermore, oral administration leads to higher levels of IL-4 compared to IFN-γ and IL-2. The host organism appears to favor a humoral immune response, although the combined numerical levels of IL-2 and IFN-γ surpass those of IL-4. This phenomenon can be attributed to the necessity of maintaining homeostasis and fulfilling an immunological function, which entails reciprocal regulation and restriction among Th1, Th2, and Th17. Th1 and Th2 cells mutually inhibit each other during immune responses to prevent excessive immune reactions. Likewise, Th17 cells are suppressed in inflammatory responses to avert excessive inflammation that may result in tissue harm. Nevertheless, the effectiveness of an oral vaccine for PEDV was validated through experimental trials conducted on mice. Despite BALB/c mice not being a susceptible animal model for PEDV, our findings indicate that the surface-displaying *L. mucosae* G01 fusion expression of the SLP chimeric immunodominant epitope 1 has the potential to be a novel mucosal vaccine. This presents an opportunity for the development of a PEDV vaccine. Unfortunately, we were unable to conduct protection trials in piglets due to the high prevalence of PEDV during the winter season and the limited availability of seronegative pigs in our study area. The presence of interference in this particular situation would have hindered the precise evaluation of the protective attributes of piglets who were orally administered with recombinant *L. mucosae* G01 fusion expressing SLP chimeric epitope 1.

Our research demonstrated the identification of B-cell linear epitopes with neutralizing capabilities through the use of bioinformatics, thereby unveiling a potentially effective mucosal vaccine candidate. In our study, we employed *L. mucosae* G01 as a vector to express exogenous SLP chimeric immunodominant epitope 1 within a surface-displaying system. The present study successfully elicited strong mucosal and humoral immune responses against PEDV in mice through oral administration, demonstrating the potential efficacy of this approach as a mucosal vaccination strategy.

## Data Availability

All data generated or analyzed during this study are included in this article.

## References

[1] Wang Q, Vlasova AN, Kenney SP, Saif LJ (2019). Emerging and re-emerging coronaviruses in pigs. Curr Opin Virol.

[2] Jung K, Saif LJ, Wang Q (2020). Porcine epidemic diarrhea virus (PEDV): An update on etiology, transmission, pathogenesis, and prevention and control. Virus Res.

[3] Liu X, Zhang L, Zhang Q, Zhou P, Fang Y, Zhao D (2019). Evaluation and comparison of immunogenicity and cross-protective efficacy of two inactivated cell culture-derived GIIa- and GIIb-genotype porcine epidemic diarrhea virus vaccines in suckling piglets. Vet Microbiol.

[4] Ji Z, Shi D, Shi H, Wang X, Chen J, Liu J (2021). A porcine epidemic diarrhea virus strain with distinct characteristics of four amino acid insertion in the COE region of spike protein. Vet Microbiol.

[5] Gerdts V, Zakhartchouk A (2017). Vaccines for porcine epidemic diarrhea virus and other swine coronaviruses. Vet Microbiol.

[6] Bae J-L, Lee J-G, Kang T-J, Jang H-S, Jang Y-S, Yang M-S (2003). Induction of antigen-specific systemic and mucosal immune responses by feeding animals transgenic plants expressing the antigen. Vaccine.

[7] Di-qiu L, Jun-wei G, Xin-yuan Q, Yan-ping J, Song-mei L, Yi-jing L (2012). High-level mucosal and systemic immune responses induced by oral administration with Lactobacillus-expressed porcine epidemic diarrhea virus (PEDV) S1 region combined with Lactobacillus-expressed N protein. Appl Microbiol Biotechnol.

[8] Bhuyan AA, Memon AM, Bhuiyan AA, Zhonghua L, Zhang B, Ye S (2018). The construction of recombinant Lactobacillus casei expressing BVDV E2 protein and its immune response in mice. J Biotechnol.

[9] Taghinezhad-S S, Keyvani H, Bermúdez-Humarán LG, Donders GGG, Fu X, Mohseni AH (2021). Twenty years of research on HPV vaccines based on genetically modified lactic acid bacteria: an overview on the gut-vagina axis. Cell Mol Life Sci.

[10] Vela Ramirez JE, Sharpe LA, Peppas NA (2017). Current state and challenges in developing oral vaccines. Adv Drug Deliv Rev.

[11] Gonzalez-Cruz P, Gill HS (2021). Demystifying particle-based oral vaccines. Expert Opin Drug Deliv.

[12] Velikova T, Snegarova V, Kukov A, Batselova H, Mihova A, Nakov R (2021). Gastrointestinal mucosal immunity and COVID-19. World J Gastroenterol.

[13] Gao X, Ma Y, Wang Z, Bai J, Jia S, Feng B (2019). Oral immunization of mice with a probiotic *Lactobacillus casei* constitutively expressing the α-toxoid induces protective immunity against *Clostridium perfringens* α-toxin. Virulence.

[14] Seegers JFML (2002). Lactobacilli as live vaccine delivery vectors: progress and prospects. Trends Biotechnol.

[15] Lin J, Zou Y, Ma C, She Q, Liang Y, Chen Z (2015). Heterologous Expression of Mannanase and Developing a New Reporter Gene System in Lactobacillus casei and Escherichia coli. PLoS ONE.

[16] Lederer FL, Günther TJ, Raff J, Pollmann KE (2011). *coli* filament formation induced by heterologous S-layer expression. Bioeng Bugs.

[17] Wang D, Liu Q, Jiang Y-L, Huang H-B, Li J-Y, Pan T-X (2021). Oral immunization with recombinant *Lactobacillus plantarum* expressing Nudix hydrolase and 43 kDa proteins confers protection against *Trichinella spiralis* in BALB/c mice. Acta Trop.

[18] Arukha AP, Freguia CF, Mishra M, Jha JK, Kariyawasam S, Fanger NA (2021). *Lactococcus lactis* Delivery of Surface Layer Protein A Protects Mice from Colitis by Re-Setting Host Immune Repertoire. Biomedicines.

[19] Uriza PJ, Trautman C, Palomino MM, Fina Martin J, Ruzal SM, Roset MS (2020). Development of an Antigen Delivery Platform Using *Lactobacillus acidophilus* Decorated With Heterologous Proteins: A Sheep in Wolf’s Clothing Story. Front Microbiol.

[20] Wang L, Zhao J, Schank M, Khanal S, Dang X, Cao D (2022). Identification of virus-specific B-cell epitopes by convalescent plasma from COVID-19 patients. Mol Immunol.

[21] Wang A, Tian Y, Liu H, Ding P, Chen Y, Liang C (2022). Identification of three conserved linear B cell epitopes on the SARS-CoV-2 spike protein. Emerg Microbes Infect.

[22] Polyiam K, Ruengjitchatchawalya M, Mekvichitsaeng P, Kaeoket K, Hoonsuwan T, Joiphaeng P (2022). Immunodominant and neutralizing linear B-Cell epitopes spanning the spike and membrane proteins of porcine epidemic diarrhea virus. Front Immunol.

[23] Chen H-Z, Tang L-L, Yu X-L, Zhou J, Chang Y-F, Wu X (2020). Bioinformatics analysis of epitope-based vaccine design against the novel SARS-CoV-2. Infect Dis Poverty.

[24] Wrapp D, McLellan JS (2019). The 3.1-Angstrom Cryo-electron Microscopy Structure of the Porcine Epidemic Diarrhea Virus Spike Protein in the Prefusion Conformation. J Virol.

[25] Kirchdoerfer RN, Bhandari M, Martini O, Sewall LM, Bangaru S, Yoon K-J (2021). Structure and immune recognition of the porcine epidemic diarrhea virus spike protein. Structure.

[26] Huang C-Y, Draczkowski P, Wang Y-S, Chang C-Y, Chien Y-C, Cheng Y-H (2022). In situ structure and dynamics of an alphacoronavirus spike protein by cryo-ET and cryo-EM. Nat Commun.

[27] Heitmann JS, Bilich T, Tandler C, Nelde A, Maringer Y, Marconato M (2022). A COVID-19 peptide vaccine for the induction of SARS-CoV-2 T cell immunity. Nature.

[28] Cañas-Arranz R, Forner M, Defaus S, De León P, Bustos MJ, Torres E (2020). A single dose of dendrimer b2t peptide vaccine partially protects pigs against foot-and-mouth disease virus infection. Vaccines.

[29] Saha S, Raghava GPS (2006). Prediction of continuous B-cell epitopes in an antigen using recurrent neural network. Proteins Struct Funct Bioinforma.

[30] Jespersen MC, Peters B, Nielsen M, Marcatili P (2017). BepiPred-2.0: improving sequence-based B-cell epitope prediction using conformational epitopes. Nucleic Acids Res.

[31] Doytchinova IA, Flower DR (2007). VaxiJen: a server for prediction of protective antigens, tumour antigens and subunit vaccines. BMC Bioinformatics.

[32] Dimitrov I, Naneva L, Doytchinova I, Bangov I (2014). AllergenFP: allergenicity prediction by descriptor fingerprints. Bioinformatics.

[33] Gupta S, Kapoor P, Chaudhary K, Gautam A, Kumar R, Open Source Drug Discovery Consortium, et al. In Silico Approach for Predicting Toxicity of Peptides and Proteins. PLoS ONE. 2013;8:e73957. doi: 10.1371/journal.pone.0073957

[34] Senior AW, Evans R, Jumper J, Kirkpatrick J, Sifre L, Green T (2020). Improved protein structure prediction using potentials from deep learning. Nature.

[35] Seeliger D, De Groot BL (2010). Ligand docking and binding site analysis with PyMOL and Autodock/Vina. J Comput Aided Mol Des.

[36] Lee J-S, Poo H, Han DP, Hong S-P, Kim K, Cho MW (2006). Mucosal Immunization with Surface-Displayed Severe Acute Respiratory Syndrome Coronavirus Spike Protein on *Lactobacillus casei* Induces Neutralizing Antibodies in Mice. J Virol.

[37] Garrote GL, Delfederico L, Bibiloni R, Abraham AG, Fernando Pérez P, Semorile L (2004). Lactobacilli isolated from kefir grains: evidence of the presence of S-layer proteins. J Dairy Res.

[38] Maier HJ, Bickerton E, editors. Coronaviruses: Methods and Protocols. New York: Springer; 2020. doi: 10.1007/978-1-0716-0900-2

[39] Baek P-S, Choi H-W, Lee S, Yoon I-J, Lee YJ, Lee DS (2016). Efficacy of an inactivated genotype 2b porcine epidemic diarrhea virus vaccine in neonatal piglets. Vet Immunol Immunopathol.

[40] Opriessnig T, Gerber PF, Shen H, De Castro AMMG, Zhang J, Chen Q (2017). Evaluation of the efficacy of a commercial inactivated genogroup 2b-based porcine epidemic diarrhea virus (PEDV) vaccine and experimental live genogroup 1b exposure against 2b challenge. Vet Res.

[41] Lee SH, Yang D-K, Kim H-H, Cho I-S (2018). Efficacy of inactivated variant porcine epidemic diarrhea virus vaccines in growing pigs. Clin Exp Vaccine Res.

[42] Hou Y, Wang Q (2019). Emerging highly virulent porcine epidemic diarrhea virus: molecular mechanisms of attenuation and rational design of live attenuated vaccines. Int J Mol Sci.

[43] Hou Y, Ke H, Kim J, Yoo D, Su Y, Boley P (2019). Engineering a Live Attenuated Porcine Epidemic Diarrhea Virus Vaccine Candidate via Inactivation of the Viral 2’- *O* -Methyltransferase and the Endocytosis Signal of the Spike Protein. J Virol.

[44] Niu X, Wang Q (2022). Prevention and control of porcine epidemic diarrhea: the development of recombination-resistant live attenuated vaccines. Viruses.

[45] de Arriba ML, Carvajal A, Pozo J, Rubio P. Mucosal and systemic isotype-speci®c antibody responses and protection in conventional pigs exposed to virulent or attenuated porcine epidemic diarrhoea virus. Vet Immunol Immunopathol. 2002; 10.1016/s0165-2427(01)00417-2 11867170

[46] Sun R-Q, Cai R-J, Chen Y-Q, Liang P-S, Chen D-K, Song C-X (2012). Outbreak of porcine epidemic diarrhea in suckling piglets. China Emerg Infect Dis.

[47] Langel SN, Paim FC, Lager KM, Vlasova AN, Saif LJ (2016). Lactogenic immunity and vaccines for porcine epidemic diarrhea virus (PEDV): Historical and current concepts. Virus Res.

[48] Cruz DJM, Kim C-J, Shin H-J (2006). Phage-displayed peptides having antigenic similarities with porcine epidemic diarrhea virus (PEDV) neutralizing epitopes. Virology.

[49] Sun D, Feng L, Shi H, Chen J, Cui X, Chen H (2008). Identification of two novel B cell epitopes on porcine epidemic diarrhea virus spike protein. Vet Microbiol.

[50] Yu R, Dong S, Chen B, Liu Y, Li F, Si F (2022). Antigenicity alternations of variant PEDV S Protein Disclosed by Linear B Cell Epitope Mapping. Viruses.

[51] Li C, Li W, Lucio De Esesarte E, Guo H, Van Den Elzen P, Aarts E, et al. Cell Attachment Domains of the Porcine Epidemic Diarrhea Virus Spike Protein Are Key Targets of Neutralizing Antibodies. J Virol. 2017;91:e00273. doi: 10.1128/JVI.00273-17 10.1128/JVI.00273-17 PMC5446644 28381581

[52] Chang C-Y, Cheng I-C, Chang Y-C, Tsai P-S, Lai S-Y, Huang Y-L (2019). Identification of neutralizing monoclonal antibodies targeting novel conformational epitopes of the porcine epidemic diarrhoea virus spike protein. Sci Rep.

[53] Sára M, Sleytr UB (2000). S-Layer Proteins. J Bacteriol.

[54] Pum D, Sleytr UB (2014). Reassembly of S-layer proteins. Nanotechnology.

[55] Herrmann J, Li P-N, Jabbarpour F, Chan ACK, Rajkovic I, Matsui T (2020). A bacterial surface layer protein exploits multistep crystallization for rapid self-assembly. Proc Natl Acad Sci.

[56] Schuster D, Küpcü S, Belton DJ, Perry CC, Stöger-Pollach M, Sleytr UB (2013). Construction of silica-enhanced S-layer protein cages. Acta Biomater.

[57] Raff J, Matys S, Suhr M, Vogel M, Günther T, Pollmann K. S-Layer-Based Nanocomposites for Industrial Applications. In: Cortajarena AL, Grove TZ, editors. Protein-Based Eng Nanostructures. Cham: Springer International Publishing; 2016 . p. 245–79. doi: 10.1007/978-3-319-39196-0_11 10.1007/978-3-319-39196-0_11 27677516

[58] Blanco-Pérez F, Papp G, Goretzki A, Möller T, Anzaghe M, Schülke S (2019). Adjuvant Allergen Fusion Proteins as Novel Tools for the Treatment of Type I Allergies. Arch Immunol Ther Exp (Warsz).

[59] Oliveira MLS, Monedero V, Miyaji EN, Leite LCC, Ho P, Parez-Marta IG (2003). Expression of *Streptococcus pneumoniae* antigens, PsaA (pneumococcal surface antigen A) and PspA (pneumococcal surface protein A) by *Lactobacillus casei*. FEMS Microbiol Lett.

[60] Padovani LS, Oliveira AMSD, Dutra BC, Costa FO, Oliveira PAD (2020). Important anatomical variations of the superior posterior alveolar artery: Studied by cone beam computed tomography. Anat Histol Embryol.

[61] Ding G, Bai J, Feng B, Wang L, Qiao X, Zhou H (2019). An EGFP-marked recombinant lactobacillus oral tetravalent vaccine constitutively expressing α, ε, β1, and β2 toxoids for *Clostridium perfringens* elicits effective anti-toxins protective immunity. Virulence.

[62] Boot HJ, Pouwels PH (1996). Expression, secretion and antigenic variation of bacterial S-layer proteins. Mol Microbiol.

[63] Zhang X, Li P, Zheng Q, Hou J (2019). Lactobacillus acidophilus S-layer protein-mediated inhibition of PEDV-induced apoptosis of Vero cells. Vet Microbiol.

[64] Prado Acosta M, Geoghegan EM, Lepenies B, Ruzal S, Kielian M, Martinez MG (2019). Surface (S) Layer Proteins of Lactobacillus acidophilus Block Virus Infection via DC-SIGN Interaction. Front Microbiol.

[65] Brandtzaeg P (1988). Immunobarriers of the mucosa of the upper respiratory and digestive pathways. Acta Otolaryngol (Stockh).

[66] Konstantinov SR, Smidt H, De Vos WM, Bruijns SCM, Singh SK, Valence F (2008). S layer protein A of *Lactobacillus acidophilus* NCFM regulates immature dendritic cell and T cell functions. Proc Natl Acad Sci.

[67] Isaacson PG (1999). Gastric MALT lymphoma: From concept to cure. Ann Oncol.

[68] Kiesewetter B, Raderer M (2020). Immunomodulatory treatment for mucosa-associated lymphoid tissue lymphoma ( MALT lymphoma). Hematol Oncol.

[69] Wang X, Wang L, Huang X, Ma S, Yu M, Shi W (2017). Oral delivery of probiotics expressing dendritic cell-targeting peptide fused with porcine epidemic diarrhea virus COE Antigen: A Promising Vaccine Strategy against PEDV. Viruses.

[70] Shi C, Cheng M, Yang X, Lu Y, Yin H, Zeng Y (2020). Probiotic Lactobacillus rhamnosus GG Promotes Mouse Gut Microbiota Diversity and T Cell Differentiation. Front Microbiol.

[71] Hwang I-C, Valeriano VD, Song JH, Pereira M, Oh JK, Han K (2023). Mucosal immunization with Lactiplantibacillus plantarum-displaying recombinant SARS-CoV-2 epitopes on the surface induces humoral and mucosal immune responses in mice. Microb Cell Factories.

[72] Romagnani S (2000). T-cell subsets (Th1 versus Th2). Ann Allergy Asthma Immunol.

[73] Quintans JSS, Shanmugam S, Heimfarth L, Araújo AAS, Almeida JRS, Picot L (2019). Monoterpenes modulating cytokines - A review. Food Chem Toxicol.

[74] Waśkiel-Burnat A, Osińska M, Salińska A, Blicharz L, Goldust M, Olszewska M (2021). The role of serum Th1, Th2, and Th17 cytokines in patients with alopecia areata: clinical implications. Cells.

